# Determinants of blood eosinophil levels in the general population and patients with COPD: a population-based, epidemiological study

**DOI:** 10.1186/s12931-022-01965-3

**Published:** 2022-03-05

**Authors:** Marc Miravitlles, Juan José Soler-Cataluña, Joan B. Soriano, Francisco García-Río, Pilar de Lucas, Inmaculada Alfageme, Ciro Casanova, José Miguel Rodríguez González-Moro, M. Guadalupe Sánchez-Herrero, Julio Ancochea, Borja G. Cosío

**Affiliations:** 1 Pneumology Department, Hospital Universitari Vall d’Hebron/Vall d’Hebron Institut de Recerca (VHIR), Vall d’Hebron Barcelona Hospital Campus, P. Vall d’Hebron 119-129, 08035 Barcelona, Spain; 2 CIBER de Enfermedades Respiratorias (CIBERES), Instituto de Salud Carlos III (ISCIII), Madrid, Spain; 3 Servicio de Neumología, Hospital Arnau de Vilanova-Lliria, Universidad de la Laguna, Valencia, Spain; 4 Servicio de Neumología, Hospital Universitario la Princesa, Universidad Autónoma de Madrid, Madrid, Spain; 5 Servicio de Neumología, Hospital Universitario la Paz-IdiPAZ, Madrid, Spain; 6 Servicio de Neumología, Hospital General Gregorio Marañon, Madrid, Spain; 7 Unidad de Gestión Clínica de Neumología, Hospital Universitario Virgen de Valme, Universidad de Sevilla, Sevilla, Spain; 8 Servicio de Neumología, Hospital Universitario Nuestra Señora de Candelaria, Tenerife, Spain; 9 Servicio de Neumología, Hospital Universitario “Príncipe de Asturias”, Alcalá de Henares, Universidad de Alcalá, Madrid, Spain; 10 Departamento Médico, GSK, Tres Cantos, Madrid, Spain; 11 Servicio de Neumología, Hospital Universitario Son Espases-IdISBa, Palma de Mallorca, Spain

**Keywords:** Epidemiology, Computed tomography, Imaging population, Biomarkers

## Abstract

**Background:**

Blood eosinophils are considered a biomarker for the treatment of chronic obstructive pulmonary disease (COPD). Population-based studies are needed to better understand the determinants of the blood eosinophil count (BEC) in individuals with and without COPD.

**Methods:**

EPISCAN II is a multicentre, cross-sectional, population-based epidemiological study aimed at investigating the prevalence and determinants of COPD in Spain. Study subjects were randomly selected from the general population, and COPD was defined by a post-bronchodilator FEV_1_/FVC < 0.7. For the pre-specified outcomes related to BEC, the first 35 COPD and 35 non-COPD subjects were consecutively recruited in 12 of the participating centres with the objective of analysing 400 individuals in each group. Baseline BEC and its association with demographic, clinical and functional variables were analysed.

**Results:**

A total of 326 COPD and 399 non-COPD subjects were included in the analysis. The mean age (standard deviation [SD]) was 63.2 years (11.0), 46.3% were male, and 27.6% were active smokers. BEC was significantly higher in individuals with COPD [192 cells/μL (SD: 125) vs. 160 cells/μL (SD: 114); p = 0.0003]. In a stepwise multivariate model, being male, active smoker and having a previous diagnosis of asthma were independently associated with having a higher BEC.

**Conclusions:**

This population-based study estimated the distribution of eosinophils in the healthy adult population and concluded that COPD patients have a significantly higher BEC. Male sex, active smoking and concomitant asthma were significantly associated with a higher BEC.

## Background

Chronic obstructive pulmonary disease (COPD) is a heterogeneous disease with multiple phenotypes and endotypes, which may be associated with different prognoses and response to treatment [1, 2]. One of the most widely studied phenotypes of COPD is that associated with increased eosinophilic inflammation. Although there is still controversy about the role of the blood eosinophil count (BEC) as a marker of risk of exacerbation [3–8], there is wide consensus about the role of the BEC as a biomarker to identify patients who will present better response to inhaled corticosteroids (ICS) [9–11]. In fact, the most recent recommendations of pharmacologic treatment include the BEC as a guide for the indication of treatment with ICS, particularly in patients at risk of exacerbations [12, 13].

In order to better understand the role of blood eosinophils as a biomarker in COPD it is important to generate new data about the distribution of blood eosinophil values in healthy adult populations and different groups of patients with COPD according to different phenotypes, degree of severity and treatments. While the distribution of blood eosinophils has been examined in several United States [3, 14] or North and Central Europe-based COPD populations [14–18], data from Southern European countries are limited.

EPISCAN II is a population-based, epidemiological study the main objective of which was to investigate the prevalence and determinants of COPD in all autonomous communities in Spain [19]. One of the pre-specified secondary objectives of the EPISCAN II study was to analyse the distribution of BECs in a subsample of participants with and without COPD in order to estimate BECs in the general adult Spanish population and to investigate factors associated with an elevated BEC [19]. The current article presents the results of the analysis of BECs and their determinants in a subgroup of participants in the EPISCAN II study with and without COPD from centres distributed throughout Spain.

## Methods

EPISCAN II is a national, multicentre, cross-sectional, population-based epidemiological study the main objective of which was to investigate the prevalence of COPD in Spain. The full protocol, the fieldwork and all the methods have been described previously [19, 20]. Briefly, 20 hospitals from the 17 Spanish autonomous communities conducted the study from April 2017 to February 2019. Subjects from the general population who were resident in the postal code areas nearest the participating hospitals were selected. A list of random telephone numbers was obtained, stratified according to these postal codes and quotas for sex and age groups. The inclusion criteria were as follows: men or women aged 40 years or more with no physical or cognitive difficulties that would prevent them from completing spirometry or any of the study procedures.

The study was approved by the ethics committees of each of the participating centres, and all participants provided informed consent. The EPISCAN II protocol is registered at https://clinicaltrials.gov (NCT03028207) and at www.gsk-clinicalstudyregister.com/study/205932.

### Variables and procedures

Demographic information on age, sex, level of education, family conditions, comorbidities, weight, height and smoking were collected. The number of respiratory exacerbations in the last 12 months was also collected. Forced spirometry was performed using a pneumotachograph (Vyntus Spiro, Carefusion, Germany), according to standardized procedures [21], and Global Lung Function Initiative equations were used as the reference value [22]. Spirometry was performed before and 15–30 min after inhalation of 400 μg of salbutamol. COPD was defined as a post-bronchodilator forced expiratory volume in one second/forced vital capacity (FEV_1_/FVC) ratio < 0.7. The study population was divided into two cohorts, depending on the results of the post-bronchodilator spirometry: patients with COPD (FEV_1_/FVC < 0.7) and non-COPD individuals (FEV_1_/FVC ≥ 0.7). Symptoms were assessed by the COPD Assessment Test (CAT) questionnaire. Physical activity was measured by the Yale Physical Activity Questionnaire (YPAS) validated for the Spanish population and the elderly population. This questionnaire reflects the amount, frequency, and intensity of physical activity that can be used to estimate the effects of physical activity as a continuous parameter, even at the low levels of activity that might be expected in COPD patients [23, 24]. The 6-min walking test (6MWT) was performed according to the American Thoracic Society (ATS) guidelines [25].

Absolute values and percentage of diffusing capacity of the lung for carbon monoxide (DLCO) and alveolar volume were collected by single-breath CO diffusing capacity (MasterScreen diffusion, Carefusion, Germany), according to the ATS/European Respiratory Society (ERS) recommendations [26]. Twenty ml of venous blood were collected from each participant for routine blood analysis, and determination of C-reactive protein (CRP) and fibrinogen. The collection procedure was standardised, and each centre stored the samples at − 80 °C until shipping for centralised analysis.

Computed tomography (CT) images were acquired during inhalation, without contrast and with low-dose radiation. The images obtained underwent semi-automatic post-processing for determination of the percentage of emphysema, areas of extension, airway thickness, and other lung parenchyma attenuation and airway wall thickness parameters [27–29].

### Statistical analysis

Considering the 10.2% prevalence of COPD found in the previous EPISCAN study [28] a priori sample size calculation estimated that with an accuracy of ± 0.6% and a 10% dropout rate, approximately 10,200 eligible individuals should be included in the study for the primary outcome, that is, the prevalence of COPD in Spain. For secondary outcomes related to CT scan and blood analysis, the first 35 COPD and 35 non-COPD subjects were consecutively invited to participate in 12 of the participating centres with the objective of recruiting a total of approximately 400 individuals in each group. Categorical variables were presented as numbers with percentages, and continuous variables as mean with standard deviation (SD). The characteristics of the population in the diagnosis of COPD groups were compared using the Student’s t-test, or χ^2^ test. The comparisons of blood eosinophil levels according to clinical, functional and CT characteristics were tested for significance using the Student’s t-test and analysis of variance. Correlations between continuous variables were evaluated using Pearson’s correlation coefficient. Univariate and multivariate linear regression analyses were used to investigate factors associated with blood eosinophil levels. Only variables with a level of significance < 0.1 in the univariate analysis were included in a stepwise multivariate linear regression model. The R2 coefficient of determination was calculated for the model. Data were analysed with the Statistical Analysis System (SAS) Enterprise Guide 7.15, considering a statistical significance (p) of 0.05 for all the statistical tests performed.

## Results

### Population

The 12 participating centres recruited a total of 326 COPD and 399 non-COPD subjects, who constituted the population of our study. The mean age of the participants was 63.2 years (SD): 11.0, and 46.3% were male, and 27.6% were active smokers. Most clinical characteristics were significantly different between COPD and non-COPD participants. COPD subjects were older, more frequently male, less frequently never smokers, with lower lung function parameters, less exercise tolerance and higher CAT scores (Table 1).

**Table 1:** Demographic, clinical, blood, and imaging characteristics of participants according to the presence of airflow limitation compatible with COPD

	COPD (n = 326)	Non-COPD (n = 399)	All (n = 725)	P value
Demographic				
Age, years	66.9 (10.5)	60.1 (10.5)	63.2 (11.0)	< 0.0001
Sex, male, %	182 (55.8%)	154 (38.6%)	336 (46.3%)	< 0.0001
BMI	27.1 (4.4)	27.1 (4.8)	27.1 (4.6)	0.986
Smoker	114 (35.0%)	86 (21.6%)	200 (27.6%)	< 0.0001
Former smoker	151 (46.3%)	152 (38.1%)	303 (41.8%)	
Never smoker	61 (18.7%)	161 (40.4%)	222 (30.6%)	
Clinical				
Asthma diagnosis	42 (12.9%)	32 (8.0%)	74 (10.2%)	0.031
CAT	9.2 (6.9)	7.0 (5.9)	8.0 (6.5)	< 0.0001
FEV_1_, L	2.2 (0.7)	2.9 (0.7)	2.6 (0.8)	< 0.0001
FEV_1_, %	82.0 (18.7)	104.6 (14.6)	94.4 (20.0)	< 0.0001
DLCO, %	89.9 (23.1)	98.8 (18.4)	94.8 (21.1)	< 0.0001
FEV_1_/FVC	62.0 (8.4)	79.8 (5.1)	71.8 (11.1)	< 0.0001
6 MWD, m	478 (108)	518 (98)	501 (105)	< 0.0001
YPAS, score	45.7 (23.1)	48.4 (21.6)	47.2 (22.3)	0.111
Exacerbations last year	0.27 (0.82)	0.05 (0.27)	0.14 (0.60)	< 0.0001
Treatment with ICS	61 (18.7%)	16 (4.0%)	77 (10.6%)	< 0.0001
Blood				
Erythrocyte, cells/µl	4.95 (0.52)	4.92 (0.44)	4.93 (0.47)	0.796
Leukocyte, cells/µl	7273 (2194)	6746 (1838)	6983 (2022)	0.0005
Neutrophils, cells/µl	4284 (1603)	3925 (1426)	4086 (1518)	0.001
Neutrophils, %	58.5 (9.2)	57.3 (8.3)	57.9 (8.8)	0.068
Eosinophils, cells/µl	192 (125)	160 (114)	175 (120)	0.0003
Eosinophils < 100 cell/μL	67 (20.6%)	131 (32.8%)	198 (27.3%)	0.0002
Eosinophils > 300 cells/μL	52 (16.0%)	46 (11.5%)	98 (13.5%)	0.080
Eosinophils, %	2.8 (1.8)	2.4 (1.6)	2.6 (1.7)	0.008
Platelets, cells/µl	233 (58)	241 (62)	238 (61)	0.070
Fibrinogen (g/L)	3.9 (0.9)	3.8 (0.9)	3.8 (0.9)	0.208
CRP (mg/dL)	2.0 (4.1)	1.5 (2.8)	1.7 (3.4)	0.0514
Imaging				
Emphysema, %	10.0 (11.9)	4.7 (7.5)	7.0 (10.0)	< 0.0001
Airway wall area, % (Primary bronchi)	18.4 (2.9)	16.9 (3.4)	17.6 (3.3)	< 0.0001
Airway wall area, % (Secondary bronchi)	27.5 (5.7)	24.6 (5.6)	25.9 (5.8)	< 0.0001

*BMI* body mass index, *CAT* COPD assessment test, *FEV1* forced expiratory volume in the first second, *FVC* forced vital capacity, *DLCO* diffusion capacity of the lung for carbon monoxide, *6MWD* 6-min walk distance, *YPAS* Yale Physical Activity Questionnaire, *CRP* C-reactive protein

Regarding blood analysis, COPD individuals had no significant differences in red blood cells, platelets, fibrinogen (g/L) or CRP (mg/dL) values but did have higher concentrations of total leukocytes, neutrophils and eosinophils (p < 0.05) (Table 1).

On CT imaging, COPD participants showed a higher percentage of areas of emphysema [10% (11.9) vs. 4.7% (7.5); p < 0.0001] and higher percentages of airway wall area in both primary and secondary bronchi (both p < 0.0001) (Table 1).

### Blood eosinophil counts according to the different subject characteristics

**Fig. 1:**
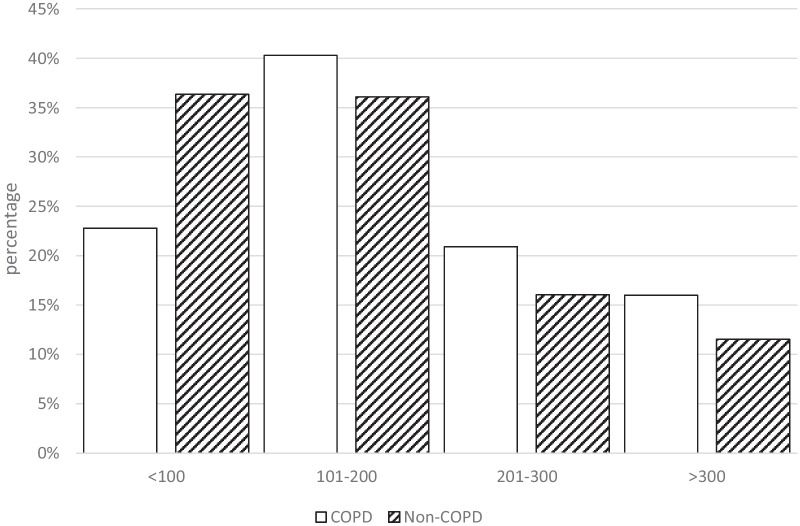
Distribution of BEC in individuals with and without COPD

The mean BEC was 175 cells/μL (SD: 120; 95% confidence interval [CI]: 166;184) and median 150 cells/μL. BECs were significantly higher in individuals with COPD (192 cells/μL (SD: 125; median: 177 cells/μL) vs. 160 cells/μL (SD: 114; median: 132 cells/μL); p = 0.0003). The distribution of blood eosinophils in both groups of subjects is depicted in Fig. 1. There were no differences in the proportion of subjects with more than 300 eosinophils/μL (11.5% in non-COPD vs. 16% in COPD, p = 0.08), but there were more non-COPD subjects with less than 100 eosinophils/μL compared with COPD patients (32.8% vs. 20.6%, p = 0.0002). In the 159 subjects with airflow obstruction according to the lower limit of normal of FEV1/FVC the mean BEC was 204 cells/μL (SD: 135; 95% confidence interval [CI]: 182;225) and median 180 cells/μL. BECs were also significantly higher in men, smokers, individuals with more advanced stages of COPD and in those with a previous diagnosis of asthma.

The differences in BEC according to CT scan parameters did not achieve statistical significance (Table 2). This analysis was repeated separately for the COPD patients and the non-COPD participants, and neither subgroup showed significant differences in BEC between individuals with or without significant emphysema or airway thickness (data not shown). Since the majority of the COPD patients identified were mild, we changed the cut off of emphysema from 10 to 5%, but again no significant differences in BEC were observed (189.5 cells/μL (SD: 134.2) in patients with 0–5% emphysema compared to 202.8 cells/μL (SD: 129.1) in patients with > 5% emphysema; p = 0.18).

**Table 2:** Blood eosinophil levels according to clinical, functional and CT characteristics

	Blood eosinophil counts		P value
	Mean (SD) (95%CI)	n	
Sex			
Male	194.8 (127.7) (181.1; 208.5)	336	< 0.0001
Female	158.1 (111.7) (147.0; 169.3)	388	
Age			
40–50	166.2 (123.4) (139.9; 192.5)	87	0.848
50–60	178.5 (126.8) (161.1; 196.1)	204	
60–70	173.0 (118.6) (157.3; 188.8)	219	
> 70	177.7 (116.4) (162.0; 193.4)	214	
BMI			
Underweight	166.8 (132.6) (44.2; 289.6)	7	0.928
Normal	171.7 (136.8) (154.4; 189.0)	243	
Overweight	178.3 (112.7) (165.6; 191.0)	306	
Obese	173.9 (108.5) (157.2; 190.6)	165	
Smoking			
Smoker	189.4 (123.2) (172.2; 206.6)	200	0.025
Former smoker	178.1 (123.3) (164.2; 192.1)	303	
Never smoker	158.2 (113.1) (143.2; 173.2)	221	
Asthma diagnosis			
Yes	204.5 (142.0) (171.6; 237.4)	74	0.027
No	171.8 (117.7) (162.8; 180.9)	650	
COPD			
No	160.7 (114.7) (149.4; 172.0)	399	0.001
GOLD I	185.8 (115.9) (168.9; 202.8)	183	
GOLD II	197.4 (133.4) (173.3; 221.5)	120	
GOLD III/IV	227.0 (158.0) (157.0; 297.1)	22	
DLCO			
> 80%	170.8 (118.5) (160.8; 180.8)	545	0.202
< 80%	184.2 (116.9) (166.2; 202.3)	163	
Exacerbations			
0	173.5 (119.2) (164.4; 182.5)	667	0.222
1 or more	195.0 (137.1) (158.6; 231.4)	57	
YPAS score			
< 51 sedentary	180.3 (122.1) (168.5; 192.0)	416	0.207
≥ 51 non-sedentary	169.2 (118.5) (155.6; 182.7)	295	
Emphysema			
0–10%	173.0 (123.7) (161.9; 184.1)	480	0.084
> 10%	190.3 (130.5) (167.9; 212.8)	132	
Airway Wall thickness (Primary Bronchi)			
0–20%	179.9 (124.3) (168.9; 191.0)	485	0.071
> 20%	164.4 (128.4) (141.9; 187.0)	127	
Airway Wall thickness (Secondary Bronchi)			
0–30%	173.9 (119.5) (163.3; 184.5)	489	0.192
> 30%	190.4 (146.0) (164.2; 216.7)	121	

*BMI* body mass index, *GOLD* Global Initiative for Obstructive Lung Disease, *COPD* chronic obstructive pulmonary disease, *YPAS* Yale Physical Activity Questionnaire, *DLCO* diffusion capacity of the lung for carbon monoxide, *CI* confidence interval, *SD* standard deviation

**Fig. 2:**
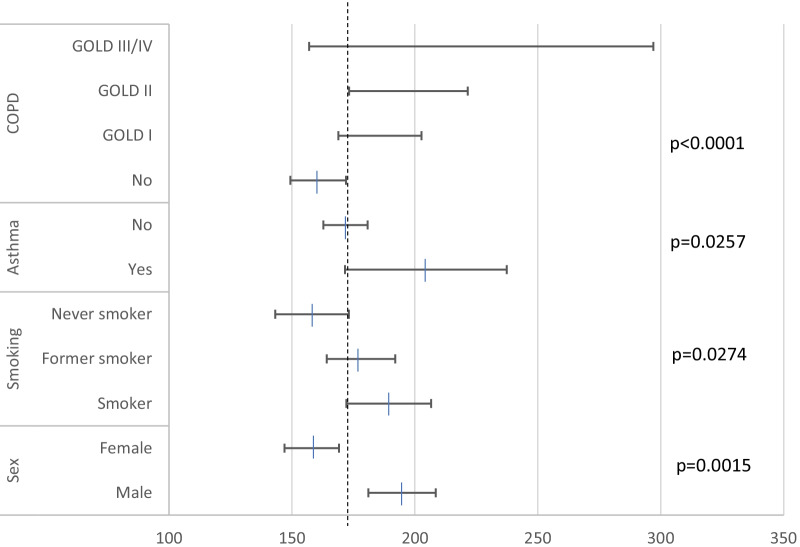
Mean and 95% confidence intervals of BEC of individuals included in the study according to different characteristics. Vertical dotted line denotes mean blood eosinophil value for the whole population

Figure 2 shows the mean and 95%CI of blood eosinophil levels in the different subgroups of participants classified according to the variables showing significant differences in univariate analysis.

### Factors independently associated with BEC

A stepwise multivariate model was developed with the following variables: age, sex, body mass index, smoking, previous diagnosis of asthma, COPD, CAT scores, FEV1 (%), DLCO (%), YPAS score, 6MWD, exacerbations, ICS treatment, platelets, fibrinogen, CRP, emphysema (% fixed threshold), and airway wall area (for central and peripheral airways in %). Only five variables had a p value < 0.10 in the final model, with three being significant at a level of < 0.05. Being male, active smoker, and having a previous diagnosis of asthma were significantly and independently associated with a higher BEC (Table 3).

**Table 3:** Multivariate analysis of the factors associated with the BEC

Parameter	Univariate	Multivariate	
	P value	Estimate (standard error)	P value
Intercept		**208.09 (31.01)**	**< 0.0001**
Age (years)	0.4555		
Sex			
Female	Reference	Reference	
Male	**< 0.0001**	**30.98 (10.65)**	**0.0038**
BMI (kg/m^2^)	0.8615		
Smoking			
Never smoker	Reference	Reference	
Former smoker	**0.0620**	10.97 (12.73)	0.3892
Active smoker	**0.0081**	**31.21 (13.68)**	**0.0229**
Asthma			
No	Reference	Reference	
Yes	**0.0274**	**36.91 (16.83)**	**0.0288**
CAT score	0.1159		
FEV1 (%)	**0.0002**	− 0.53 (0.28)	0.0543
DLCO (%)	**0.0474**		
YPAS score	**0.0493**		
6 MWD, m	0.3057		
Exacerbations last year	**0.0331**		
COPD			
No	Reference		
Yes	**0.0003**		
Treatment with ICS	**0.0099**		
Platelets, cells/µl	0.1026		
Fibrinogen (g/L)	0.9357		
CRP (mg/dL)	**0.0831**		
Total emphysema volume (%)	**0.0082**	0.94 (0.51)	0.0660
% Airway wall area (Primary Bronchi)	0.4094		
% Airway wall area (Secondary bronchi)	0.1527		

*BMI* body mass index, *CAT* COPD assessment test, *FEV1* forced expiratory volume in the first second, *FVC* forced vital capacity, *DLCO* diffusion capacity of the lung for carbon monoxide, *6MWD* 6-min walk distance, *YPAS* Yale Physical Activity Questionnaire, *CRP* C-reactive protein. Values in bold are statistically significant. R-Square = 0.067

## Discussion

Given the potential for BEC as a biomarker in COPD [9, 11, 30], it is important to understand the distribution and determinants of BEC in the general adult population and in COPD patients. Our population-based sample of non-COPD adults recruited in the EPISCAN II study had a mean BEC of 160 cells/μL, being significantly higher for COPD patients. We also observed a significantly higher BEC in males, active smokers, and individuals with previous history of asthma and increased levels of eosinophils with increasing severity of airflow obstruction.

Since the EPISCAN II was a population-based epidemiological study, it is important to note that the majority of our population of COPD patients had mild disease, with 56% of GOLD stage I, and 75% were undiagnosed and untreated [20].

The mean BEC in non-COPD subjects was 160 cells/μL, which was significantly lower than the mean count in COPD patients (192 cells/μL). The concentrations observed in our population of non-COPD individuals were intermediate compared to those observed in an Austrian population described by Hartl et al*.* [17] with a geometric mean of 128 cells/μL, or those reported in a study in the UK (140 cells/μL in smokers and 120 cells/μL in non-smokers) [18], and the concentrations observed in the UK Clinical Practice Research Datalink (182 cells/μL) [15]. However, they were very similar to the values reported in Copenhagen, with a median value of 150 cells/μL in adult never smokers and 160 cells/μL in ever smokers [16].

Our data replicate previous results showing a significantly higher BEC in subjects with COPD compared with non-COPD controls. In the previously mentioned study by Kolsum et al*.* [18], the mean BEC was 209 cells/μL in COPD subjects, and in the UK database study, blood eosinophils were a mean of 8% higher in COPD compared with non-COPD subjects [15]. A previous population-based database study in Spain in + 57,000 patients with COPD reported a mean eosinophil count of 253 cells/μL [5], with 28.4% of individuals with counts > 300 eosinophils/μL, being clearly higher than the 192 cells/μL observed in our study with only 16% with BEC > 300 cells/μL. Similarly, in other Spanish cohorts, Cosio et al. [31] found a mean BEC of 240 cells/μL in a sample of 706 COPD patients without asthma-COPD overlap, and Soler-Cataluña et al*.* [8] reported a mean BEC of 256 cells/μL in 233 COPD patients, 38.2% with ≥ 300 eosinophils/μL, while the mean BEC in asthmatic patients was 402 cells/μL. These higher values may be explained by the different characteristics of the participants, since the subjects in these studies had diagnosed COPD with a mean FEV1(%) of between 57 and 64%, and 74% to 85% were men [5, 8, 30] compared with a mean FEV_1_(%) of 82% and 56% males in our population. Actually, some of these characteristics have been associated with BEC; in our study, male sex, active smoking, previous history of asthma and impaired FEV_1_(%) were significantly and independently associated with a higher BEC. Moreover, in previous studies, male sex [16, 17], active smoking [17, 18] and asthma diagnosis [15, 17] have been described as being associated with a higher BEC. Other factors associated with increased eosinophils described in large database study are: age < 18 years, positive skin prick test, atopy, a positive bronchodilator test, metabolic syndrome and obesity [16, 17]. Our study found not only a significant difference in BEC between COPD and non-COPD individuals, but also an increase in BEC from non-COPD to GOLD stage III/IV COPD, and similarly, a significant negative correlation between eosinophils and FEV_1_(%) indicating higher levels in more severe disease.

Interestingly, we did not observe any significant association between blood eosinophils and exacerbations or ICS treatment, probably due to the characteristics of our population which was mainly made up of mild and untreated patients. Our results concur with those of a previous large database study in patients at low risk of exacerbations in Primary Care that did not observe any influence of treatment with ICS on BEC, and no association was found between blood eosinophils and the frequency of exacerbations [5].

Blood eosinophils were not associated with percent airway wall area for either the central or peripheral airways. Apparently, there was a trend towards a higher BEC in patients with COPD and more emphysema on CT, but the differences were not significant. On the contrary, Papaioannou et al*.* [32] found that emphysema was associated with low blood eosinophil counts in a group of 98 patients with COPD. Again, these differences may be due to the different characteristics of the populations analysed; they defined emphysema as the presence of emphysema lesions in > 15% of the pulmonary parenchyma, and their emphysema patients had a mean FEV_1_(%) of 43%. In contrast, there was an underrepresentation of patients with moderate and severe COPD in our sample, and the mean percent emphysema area was 10% in our COPD patients. A recent Canadian study described that patients with COPD and an elevated BEC had thickened central airway walls and a reduction in the total number of visible airways, indicating airway remodelling [33]. More studies are needed to clarify the relationship between BEC and the classic phenotypes of chronic bronchitis and emphysema in COPD.

Our study has several limitations, since it was a population-based study of the general population, patients identified with COPD were predominantly mild, undiagnosed and paucisymptomatic, with underrepresentation of patients with other degrees of severity. However, despite these characteristics, there were clear and significant differences in their characteristics compared with non-COPD individuals, including a significantly higher concentration of blood eosinophils. The cross-sectional design did not allow evaluation of the possible prognostic value of BEC for outcomes such as exacerbations. However, the design of the study had the strength to allow the analysis of an unselected population of COPD and non-COPD individuals from all geographic areas of Spain. Finally, we did not systematically assess the presence of possible causes of hypereosinophilia, although they have a low prevalence in the general population.

## Conclusions

The results of our population-based study provide an estimate of the distribution of eosinophils in the healthy adult population in Spain and has demonstrated that COPD patients, even with milder stages, have a significantly higher BEC. In addition to COPD, male sex, active smoking, the presence of asthma and a worse FEV_1_(%) are significantly associated with a higher BEC.

## Data Availability

Information on the GSK data sharing commitments and requesting access to anonymized individual participant data and associated documents can be found at www.clinicalstudydatarequest.com.

## Abbreviations

ATS: American Thoracic Society
BEC: Blood eosinophil count
CAT: COPD assessment test
COPD: Chronic obstructive pulmonary disease
CT: Computed tomography
CRP: C-reactive protein
DLCO: Diffusing capacity of the lung for carbon monoxide
EPISCAN: Epidemiological study of COPD in Spain
ERS: European Respiratory Society
FEV1: Forced expiratory volume in 1 s
FVC: Forced vital capacity
GOLD: Global initiative for obstructive lung disease
ICS: Inhaled corticosteroids
SAS: Statistical analysis system
SD: Standard deviation
6MWT: Six-minute walking test
UK: United Kingdom
YPAD: Yale Physical Activity Questionnaire

## References

[1] Cosio BG, Soriano JB, López-Campos JL, Calle M, Soler JJ, de Torres JP (2016). Distribution and outcomes of a phenotype-based Approach to guide COPD management: results from the CHAIN cohort. PLoS ONE.

[2] Miravitlles M, Calle M, Soler-Cataluña JJ (2012). Clinical phenotypes of COPD. Identification, definition and implications for guidelines. Arch Bronconeumol.

[3] Yun JH, Lamb A, Chase R, Singh D, Parker MM, Saferali A (2018). Blood eosinophil count thresholds and exacerbations in patients with chronic obstructive pulmonary disease. J Allergy Clin Immunol.

[4] Kerkhof M, Chaudhry I, Pavord ID, Miravitlles M, Kook Rhee C, Halpin DMG (2020). Blood eosinophil count predicts treatment failure and hospital readmission for COPD. ERJ Open Res.

[5] Miravitlles M, Monteagudo M, Solntseva I, Alcázar B (2021). Blood eosinophil counts and their variability and risk of exacerbations in COPD: a population-based study. Arch Bronconeumol.

[6] Golpe R, Dacal D, Sanjuán-López P, Martín-Robles I, Pérez-de-Llano LA (2020). Plasma eosinophil count and patient-centered events in chronic obstructive pulmonary disease in real-life clinical practice. Arch Bronconeumol.

[7] Singh D, Wedzicha JA, Siddiqui S, de la Hoz A, Xue W, Magnussen H (2020). Blood eosinophils as a biomarker of future COPD exacerbation risk: pooled data from 11 clinical trials. Respir Res.

[8] Soler-Cataluña JJ, Novella L, Soler C, Nieto ML, Esteban V, Sánchez-Toril F (2020). Clinical characteristics and risk of exacerbations associated with different diagnostic criteria of asthma-COPD overlap. Arch Bronconeumol.

[9] Pavord ID, Lettis S, Locantore N, Pascoe S, Jones PW, Wedzicha JA (2016). Blood eosinophils and inhaled corticosteroid/long-acting β-2 agonist efficacy in COPD. Thorax.

[10] Brusselle G, Pavord ID, Landis S, Pascoe S, Lettis S, Morjaria N (2018). Blood eosinophil levels as a biomarker in COPD. Respir Med.

[11] Singh D, Bafadhel M, Brightling CE, Sciurba FC, Curtis JL, Martinez FJ (2020). Blood eosinophil counts in clinical trials for chronic obstructive pulmonary disease. Am J Respir Crit Care Med.

[12] Global Strategy For The Diagnosis, Management, And Prevention Of Chronic Obstructive Pulmonary Disease. 2021 Report. https://goldcopd.org/wp-content/uploads/2020/11/GOLD-REPORT-2021-v1.1-25Nov20_WMV.pdf [Accessed May 16th, 2021.

[13] Miravitlles M, Calle M, Molina J, Almagro P, Gómez JT, Trigueros JA (2021). Spanish COPD Guidelines (GesEPOC) 2021: updated pharmacological treatment of stable COPD. Arch Bronconeumol.

[14] Vogelmeier CF, Kostikas K, Fang J, Tian H, Jones B, Morgan CL (2019). Evaluation of exacerbations and blood eosinophils in UK and US COPD populations. Respir Res.

[15] Landis S, Suruki R, Maskell J, Bornar K, Hilton E, Compton C (2018). Demographic and clinical characteristics of COPD patients at different blood eosinophil levels in the UK clinical practice research datalink. COPD.

[16] Çolak Y, Afzal S, Nordestgaard BG, Marott JL, Lange P (2018). Combined value of exhaled nitric oxide and blood eosinophils in chronic airway disease: the Copenhagen General Population Study. Eur Respir J.

[17] Hartl S, Breyer MK, Burghuber OC, Ofenheimer A, Schrott A, Urban MH (2020). Blood eosinophil count in the general population: typical values and potential confounders. Eur Respir J.

[18] Kolsum U, Southworth T, Jackson N, Singh D (2019). Blood eosinophil counts in COPD patients compared to controls. Eur Respir J.

[19] Alfageme I, de Lucas P, Ancochea J, Miravitlles M, Soler-Cataluña JJ, García-Río F (2019). 10 Years After EPISCAN: a new study on the prevalence of COPD in Spain—a summary of the EPISCAN II protocol. Arch Bronconeumol.

[20] Soriano JB, Alfageme I, Miravitlles M, de Lucas P, Soler-Cataluña JJ, García-Río F (2021). Prevalence and Determinants of COPD in Spain: EPISCAN II. Arch Bronconeumol.

[21] Miller MR, Hankinson J, Brusasco V, Burgos F, Casaburi R, Coates A (2005). ATS/ERS Task Force. Standardization of spirometry. Eur Respir J.

[22] Quanjer PH, Stanojevic S, Cole TJ, Baur X, Hall GL, Culver BH, ERS Global Lung Function Initiative (2012). Multi-ethnic reference values for spirometry for the 3–95-yr age range: the global lung function 2012 equations. Eur Respir J.

[23] De Abajo S, Larriba R, Márquez S (2001). Validity and reliability of the Yale Physical Activity Survey in Spanish elderly. J Sports Med Phys Fitness.

[24] Donaire-González D, Gimeno-Santos E, Serra I, Roca J, Balcells E, Rodríguez E (2011). Validación del cuestionario de actividad física de Yale en pacientes con enfermedad pulmonar obstructiva crónica. Arch Bronconeumol.

[25] American Thoracic Society ATS Statement: Guidelines for the Six-Minute Walk Test. Am J Respir Crit Care Med 2002; 166: 111–117. 10.1164/ajrccm.166.1.at1102 12091180

[26] MacIntyre N, Crapo RO, Viegi G, Johnson DC, van der Grinten CPM, Brusasco V (2005). Standardisation of the single-breath determination of carbon monoxide uptake in the lung. Eur Respir J.

[27] Owrangi AM, Etemad-Rezai R, McCormack DG, Cunningham IA, Parraga G (2013). Computed tomography density histogram analysis to evaluate pulmonary emphysema in ex-smokers. Acad Radiol.

[28] Galban C, Han M, Boes J, Chughtai K, Meyer C, Johnson T (2012). Computed tomography-based biomarker provides unique signature for diagnosis of COPD phenotypes and disease progression. Nat Med.

[29] Nambu A, Zach J, Schroeder J, Jin G, Kim SS, Kim YI (2016). Quantitative computed tomography measurements to evaluate airway disease in chronic obstructive pulmonary disease: relationship to physiological measurements, clinical index and visual assessment of airway disease. Eur J Radiol.

[30] Bafadhel M, Pavord ID, Russell REK (2017). Eosinophils in COPD: just another biomarker?. Lancet Respir Med.

[31] Cosio BG, Soriano JB, López-Campos JL, Calle-Rubio M, Soler-Cataluna JJ, de Torres JP (2016). Defining the asthma-COPD overlap syndrome in a COPD cohort. Chest.

[32] Papaioannou AI, Kostikas K, Papaporfyriou A, Angelakis L, Papathanasiou E, Hillas G (2017). Emphysematous phenotype is characterized by low blood eosinophils: a cross-sectional study. COPD.

[33] Tan WC, Bourbeau J, Nadeau G, Wang W, Barnes N, Landis SH (2021). High eosinophil counts predict decline in FEV1: results from the CanCOLD study. Eur Respir J.

